# Modeling lactate threshold in young squad athletes: influence of sex, maximal oxygen uptake, and cost of running

**DOI:** 10.1007/s00421-022-05084-1

**Published:** 2022-11-21

**Authors:** Sanghyeon Ji, Sebastian Keller, Lukas Zwingmann, Patrick Wahl

**Affiliations:** 1 Department of Exercise Physiology, German Sport University, Cologne, Germany; 2 German Research Centre of Elite Sport, German Sport University, Cologne, Germany; 3 Department of Molecular and Cellular Sports Medicine, Institute of Cardiovascular Research and Sports Medicine, German Sport University, Cologne, Germany

**Keywords:** Maximal metabolic steady state, Performance diagnostics, Aerobic capacity, Endurance performance, Running economy, Youth athletes

## Abstract

**Purpose:**

This study aimed to investigate: 1. The influence of sex and age on the accuracy of the classical model of endurance performance, including maximal oxygen uptake ($\dot{V}\mathrm{O}_{2}\mathrm{peak}$), its fraction (LT2_%_), and cost of running (*C*_R_), for calculating running speed at lactate threshold 2 (vLT2) in young athletes. 2. The impact of different *C*_R_ determination methods on the accuracy of the model. 3. The contributions of $\dot{V}\mathrm{O}_{2}\mathrm{peak}$, LT2_%_, and *C*_R_ to vLT2 in different sexes.

**Methods:**

45 male and 55 female young squad athletes from different sports (age: 15.4 ± 1.3 years; $$\dot{V}\mathrm{O}_{2}\mathrm{peak}$$: 51.4 ± 6.8 $$\hbox {mL} \cdot \hbox {kg}^{-1} \cdot \hbox {min}^{-1}$$) performed an incremental treadmill test to determine $$\dot{V}\mathrm{O}_{2}\mathrm{peak}$$, LT2_%_, *C*_R_, and vLT2. *C*_R_ was assessed at a fixed running speed (2.8 $$ \hbox {m} \cdot \hbox {s}^{-1}
$$), at lactate threshold 1 (LT1), and at 80% of $$\dot{V}\mathrm{O}_{2}\mathrm{peak}$$, respectively.

**Results:**

Experimentally determined and modeled vLT2 were highly consistent independent of sex and age (ICC $\ge$ 0.959). The accuracy of vLT2 modeling was improved by reducing random variation using individualized *C*_R_ at 80% $\dot{V}\mathrm{O}_{2}\mathrm{peak}$ (± 4%) compared to *C*_R_ at LT1 (± 7%) and at a fixed speed (± 8%). 97% of the total variance of vLT2 was explained by $\dot{V}\mathrm{O}_{2}\mathrm{peak}$, LT2_%_, and *C*_R_. While $\dot{V}\mathrm{O}_{2}\mathrm{peak}$ and *C*_R_ showed the highest unique (96.5% and 31.9% of total $R^2$, respectively) and common (– 31.6%) contributions to the regression model, LT2_%_ made the smallest contribution (7.5%).

**Conclusion:**

Our findings indicate: 1. High accuracy of the classical model of endurance performance in calculating vLT2 in young athletes independent of age and sex. 2. The importance of work rate selection in determining *C*_R_ to accurately predict vLT2. 3. The largest contribution of $\dot{V}\mathrm{O}_{2}\mathrm{peak}$ and *C*_R_ to vLT2, the latter being more important in female athletes than in males, and the least contribution of LT2_%_.

## Introduction

Endurance performance depends on a complex interplay of various metabolic and mechanical determinants (Joyner and Coyle 2008). While aerobic capacity, i.e., maximal oxygen uptake ($\dot{V}\mathrm{O}_{2}\text{peak}$) and its fraction at a disproportionate increase in the speed-lactate curve that can be sustained over a longer period (LT2_%_) (Farrell et al. 1979; McLaughlin et al. 2010; Støa et al. 2020) represent the main metabolic determinants, movement economy (or energy cost of movement [*C*]) depends on the proportion of mechanical power output that contributes to progression (the higher the proportion, the more economical the locomotion) (Minetti 2004). Together, these three parameters have been shown to accurately predict endurance performance according to formula (1) (Joyner 1991; McLaughlin et al. 2010). For example, McLaughlin et al. (2010) found that in well-trained distance runners, 95.4% of the variation in 16-km running time could be explained by these variables.systematic analysis of validit

1 $$\begin{aligned} \text {Endurance performance}\ {=}\ \mathrm{LT2}_{\%} \cdot \frac{\dot{V}\mathrm{O}_{2}\mathrm{peak}}{C}. \end{aligned}$$

Recently, Støren et al. (2014) and Støa et al. (2020) used the same equation to model the work rate corresponding to lactate threshold 2 (LT2) in cycling and running, respectively. Representing the highest work rate that still elicits a metabolic steady state, LT2 depicts an important parameter for endurance exercise prescription (indicating the upper boundary of the heavy intensity domain) and performance prediction (showing high correlations with endurance performance) (Faude et al. 2009). Interestingly, in both disciplines, Støren et al. (2014) and Støa et al. (2020) found a strong dependence of LT2 on $\dot{V}\mathrm{O}_{2}\mathrm{peak}$ and *C* as well as a high agreement between calculated and measured work rates corresponding to LT2. Based on these observations, they concluded that training prescription may focus either on improving $\dot{V}\mathrm{O}_{2}\mathrm{peak}$ for example using high intensity interval training or on improving *C* for example by implementing maximal strength training (Støa et al. 2020). Therefore, this model could represent a way to individualize exercise prescription for endurance training.

So far, however, the model applies only to adult well-trained to elite cyclists (Støren et al. 2014) and to adult recreational to elite long-distance runners (Støa et al. 2020), but not to other sport disciplines or age groups. Especially for young well-trained athletes, the model could represent an option to individualize training prescriptions based on the physiological profiles and thus use limited training time as efficiently as possible (Støa et al. 2020). However, it must first be investigated whether the model is dependent on age, since only adult athletes have been studied so far (Støa et al. 2020; Støren et al. 2014). Further, potential differences between sexes need to be considered, as sex specific prerequisites such as body composition could influence physiological characteristics such as aerobic capacity (Besson et al. 2022). In addition, the influence of the methods used to calculate *C* (model predictor) and LT2 (criterion) are unknown. With regard to LT2, there are a large number of studies that have investigated the agreement of different methods to determine LT2 with the underlying physiological concept of a maximal metabolic steady state but have yielded heterogeneous results [e.g., Faude et al. (2009)]. Regarding the LT2 determination method used by Støren et al. (2014) and Støa et al. (2020) (i.e., warm-up blood lactate concentration + 2.3 $\hbox {mmol} \cdot \hbox {L}^{-1}$), to the best of our knowledge, no systematic analysis of validity showing the absolute level of agreement with maximal metabolic steady state has been published. Therefore, re-calculating the model with a threshold concept that validly represents maximal metabolic steady state seems warranted.

Regarding model predictors, $\dot{V}\mathrm{O}_{2}\mathrm{peak}$ and LT2_%_ represent physiologically well-defined constructs, albeit dependent on test protocol and determination method (Faude et al. 2009; Midgley et al. 2007), whereas determination of cost of running (*C*_R_) is still strongly debated (Barnes and Kilding 2015; Lundby et al. 2017). This controversy is mainly related to the question of whether or not *C*_R_ is independent of the running speed and therefore different external work rates have been studied (Iaia et al. 2009; Jones and Doust 1996; Lacour and Bourdin 2015; Svedenhag and Sjödin 1994). In contrast to the common approach of measuring oxygen uptake ($\dot{V}\mathrm{O}_{2}$) at a fixed submaximal speed (e.g., 12 $\hbox {km} \cdot \hbox {h}^{-1}$) to ensure achievement of a metabolic steady-state (Barnes and Kilding 2015), Støa et al. (2020) assessed $\dot{V}\mathrm{O}_{2}$ at a fix percentage (i.e., 70%) of $\dot{V}\mathrm{O}_{2}\mathrm{peak}$ calculated from the linear relationship between submaximal running speeds and the corresponding $\dot{V}\mathrm{O}_{2}$ values. However, apart from the fact that the linearity of the $\dot{V}\mathrm{O}_{2}$ response to exercise is controversial (DiMenna and Jones 2009), from a physiological point of view both assessment methods bear the risk to obtain heterogeneous metabolic responses due to inter-individual variability. For example, Scharhag-Rosenberger et al. (2010) reported a large variability in blood lactate response at a work rate corresponding to 60% and 75% of $\dot{V}\mathrm{O}_{2}\mathrm{peak}$. Similarly, measuring $\dot{V}\mathrm{O}_{2}$ at a fixed submaximal speed (e.g., 12 $\hbox {km} \cdot \hbox {h}^{-1}$) will most likely elicit different metabolic responses in differently trained individuals. Since such divergent internal metabolic responses may impact inter-individual comparability, assessing *C*_R_ at a distinct submaximal metabolic anchor such as the first rise in blood lactate levels (LT1) might be a more individualized option.

Due to the potential impact of the cited methodological aspects as well as participant characteristics including sporting background, age, and sex on the accuracy of the model, the aims of the present study were to investigate: 1. The accuracy of the model, originally applied to adult runners by Støa et al. (2020), in young athletes of different disciplines depending on age and sex. In contrast to Støa et al. (2020), a validated threshold concept was used as a criterion. 2. The impact of different methods to determine *C*_R_ on the accuracy of the model. 3. The influence of LT2_%_, $\dot{V}\mathrm{O}_{2}\mathrm{peak}$, and *C*_R_ on the running speed at LT2 (vLT2) in young athletes depending on sex.

## Materials and methods

### Participants

The study sample consisted of young squad athletes from the federal state of North Rhine-Westphalia, Germany (*n* = 248). All of them participated in regular training and official competitions in various sports (including endurance type individual sports and team, racket, as well as combat sports) on regional to national levels. All athletes gave their assent, and informed consent was obtained from their parents or legal guardians. The experimental procedures were approved by the local ethics committee (approval number 67/2020) and was conducted in accordance with the Declaration of Helsinki.

**Fig. 1:**
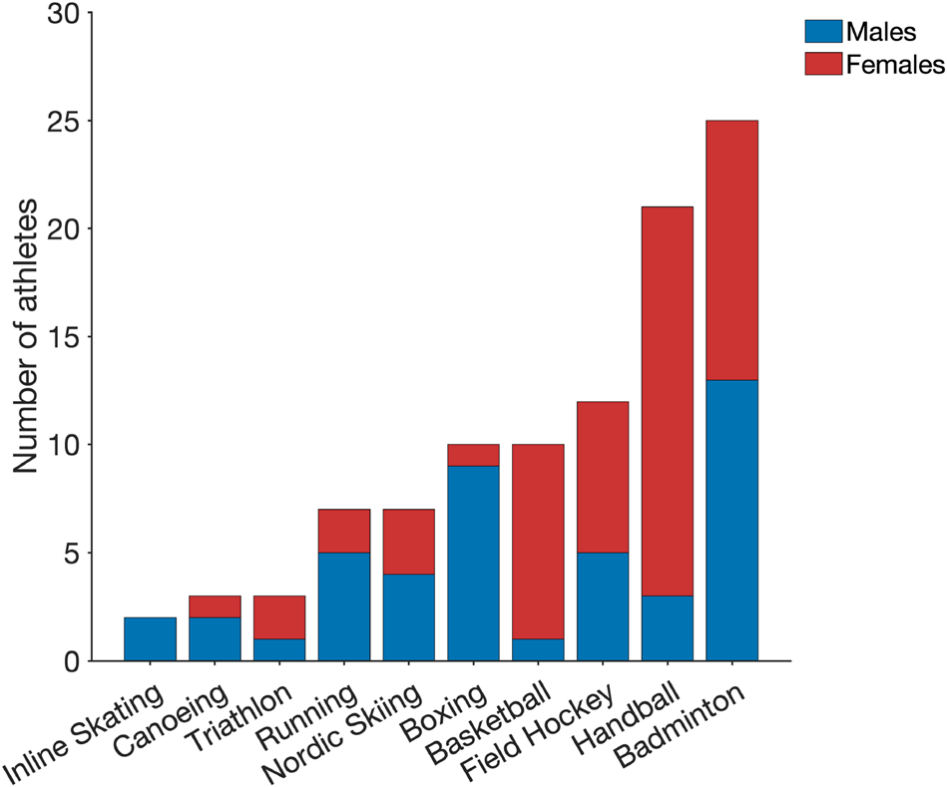
Number of athletes included in the study, separated by sex and sport discipline

To ensure validity and comparability, only data that met the following criteria were included for further analysis: (a) age < 19 years; (b) exhaustion (see below for determination of $\dot{V}\mathrm{O}_{2}\mathrm{peak}$); (c) valid determination of LT1 and LT2 using the modified $\hbox {maximal deviation}$ method (see below); (d) number of stages completed during the incremental step test > three (e) running speed at 80% of $\dot{V}\mathrm{O}_{2}\mathrm{peak}$ $\ge$ 2.4 $\hbox {m} \cdot \hbox {s}^{-1}$ (i.e., within the speed range used in the incremental test). If athletes had multiple performance diagnostic visits, only data from the first visit were used. A total of 100 athletes (45 males and 55 females) were finally included in the present study. The athlete’s anthropometric characteristics and disciplines are presented in Table 1 and Fig. 1, respectively.

**Table 1:** Descriptive anthropometric and physiological characteristics (mean ± standard deviation) of the participants

Variable	All (*N* = 100)	Males (*N* = 45)	Females (*N* = 55)	*p* (m vs. f)
Anthropometrics				
Age [y]	15.4 ± 1.3	15.8 ± 1.4	15.2 ± 1.1	0.013
Height [cm]	172.8 ± 9.1	175.2 ± 10.5	170.9 ± 7.3	0.019
Body mass [kg]	62.3 ± 11.8	63.6 ± 14.6	61.2 ± 8.9	0.303
$$\dot{V}\mathrm{O}_{2}\mathrm{peak}$$				
[$\hbox {mL} \cdot \hbox {min}^{-1}$]	3182 ± 639	3561 ± 735	2871 ± 304	< 0.001
[$\hbox {mL} \cdot \hbox {kg}^{-1} \cdot \hbox {min}^{-1}$]	51.4 ± 6.8	56.4 ± 5.9	47.3 ± 4.3	< 0.001
Oxygen *C*_R_				
*C*_R_fix [mL · kg$^{-1}$ · m$^{-1}$]	0.223 ± 0.020	0.232 ± 0.021	0.216 ± 0.017	< 0.001
*C*_R_LT1 [mL · kg$^{-1}$ · m$^{-1}$]	0.222 ± 0.020	0.230 ± 0.020	0.216 ± 0.018	< 0.001
*C*_R_80% [mL · kg$^{-1}$ · m$^{-1}$]	0.222 ± 0.018	0.229 ± 0.018	0.216 ± 0.016	< 0.001
Energy *C*_R_				
*C*_R_fix[$\hbox {J} \cdot \hbox {kg}^{-1} \cdot \hbox {m}^{-1}$]	4.84 ± 0.44	5.02 ± 0.46	4.70 ± 0.37	< 0.001
*C*_R_LT1 [$\hbox {J} \cdot \hbox {kg}^{-1} \cdot \hbox {m}^{-1}$]	4.82 ± 0.43	4.98 ± 0.43	4.68 ± 0.38	< 0.001
*C*_R_80% [$\hbox {J} \cdot \hbox {kg}^{-1} \cdot \hbox {m}^{-1}$]	4.84 ± 0.40	5.00 ± 0.39	4.71 ± 0.35	< 0.001
Lactate threshold				
LT2_%_ [%]	87.0 ± 2.6	87.0 ± 2.9	87.0 ± 2.4	0.999
vLT2 [$\hbox {m} \cdot \hbox {s}^{-1}$]	3.38 ± 0.39	3.61 ± 0.41	3.19 ± 0.24	< 0.001
_cal_LT2_fix_ [$\hbox {m} \cdot \hbox {s}^{-1}$]	3.34 ± 0.38	3.54 ± 0.42	3.18 ± 0.25	< 0.001
_cal_LT2_LT1_ [$\hbox {m} \cdot \hbox {s}^{-1}$]	3.36 ± 0.41	3.58 ± 0.44	3.18 ± 0.27	< 0.001
_cal_LT2_80%_ [$\hbox {m} \cdot \hbox {s}^{-1}$]	3.36 ± 0.39	3.58 ± 0.43	3.18 ± 0.24	< 0.001

$\dot{V}\mathrm{O}_{2}\mathrm{peak}$ maximal oxygen uptake, $C_{\mathrm{R}}$ cost of running, $C_{\mathrm{R}}\mathrm{fix}$ $C_\mathrm{R}$ determined at a fixed speed of 2.8 $\hbox {m} \cdot \hbox {s}^{-1}$, $C_{R}\mathrm{LT1}$ $C_\mathrm{R}$ determined at lactate threshold 1, $C_{\mathrm{R}}\mathrm{80\%}$ $C_{\mathrm{R}}$ determined at 80% of $\dot{V}\mathrm{O}_{2}\mathrm{peak}$, $\mathrm{LT2_{\%}}$ fractional utilization of $\dot{V}\mathrm{O}_{2}\mathrm{peak}$, *vLT2* running speed at lactate threshold 2, _*cal*_LT2_fix_ calculated vLT2 determined using *C*_R_fix, _*cal*_LT2_LT1_ calculated vLT2 determined using *C*_R_LT1, _*cal*_LT2_80%_ calculated vLT2 determined using *C*_R_80%

### Procedures

In this cross-sectional study, all athletes completed an incremental step test to determine endurance performance as part of a larger performance check-up for young squad athletes at a local performance diagnostic center between January 2018 and January 2022.

All tests were performed under constant laboratory conditions on a treadmill (h/p/cosmos, saturn^®^ 250/100, Traunstein, Germany) with an incline of 1% simulating air resistance. Following a two-minute resting measurement in standing position, the initial speed was set to 2.4 $\hbox {m} \cdot \hbox {s}^{-1}$ and increased by 0.4 $\hbox {m} \cdot \hbox {s}^{-1}$ every 5 min to ensure attainment of metabolic steady state conditions. Between the stages, short resting periods (30 s) were allowed for capillary blood sampling (20 $\upmu {\hbox {L}}$) and tests were performed until volitional exhaustion.

Throughout the test, breathing gases (Metalyzer^®^3B; Cortex Biophysik GmbH, Leipzig, Germany) and heart rate (Polar H7 Sensor; Polar Electro, Kempele, Finland) were recorded every second and averaged over 30 s. The spirometer was calibrated weekly with a reference gas (5% CO_2_ and 15% O_2_) and before each test with ambient air and with a 3-L syringe, according to the manufacturer’s specifications. Immediately after the test, blood lactate concentrations were determined (Biosen C-line; EKF Diagnostic Sales, Magdeburg, Germany).

### Parameters

Blood lactate concentrations during the incremental step test were plotted against running speed and then fitted by a third-order polynomial function. vLT2 was identified as the point on the lactate performance curve that yielded the maximal perpendicular distance to a straight line formed by the peak lactate point and by the point of the first rise in blood lactate concentration at which the slope of the fitted lactate curve equaled 1.00 (LT1). This method has recently been shown to be a valid estimate of maximal lactate steady state in running (Zwingmann et al. 2019).

$\dot{V}\mathrm{O}_{2}\mathrm{peak}$ corresponded to the highest measured 30-s moving average of $\dot{V}\mathrm{O}_{2}$ during the test. Exhaustion was verified using the following criteria (Midgley et al. 2007): respiratory exchange ratio $\ge$ 1.10, heart rate $\ge$ 95% of age predicted maximum, blood lactate concentration $\ge$ 8 mmol · L^–1^, and volitional exhaustion.

All spirometric data were averaged over the last third of each stage to ensure that a steady state was achieved at least in the submaximal stages (i.e., $\le$ vLT2) (Whipp and Wasserman 1972).

LT2_%_ was determined by dividing $\dot{V}\mathrm{O}_{2}$ corresponding to LT2 by $\dot{V}\mathrm{O}_{2}\mathrm{peak}$.

To determine *C*_R_, $\dot{V}\mathrm{O}_{2}$ at three different work rates was divided by the respective running speeds: (1) a fixed running speed of 2.8 $\hbox {m} \cdot \hbox {s}^{-1}$ (*C*_R_fix); (2) the running speed corresponding to LT1 (*C*_R_LT1); and (3) the running speed corresponding to 80% of $\dot{V}\mathrm{O}_{2}\mathrm{peak}$ as calculated from the linear regression from running speed and $\dot{V}\mathrm{O}_{2}$ ($R^2$ $\ge$ 0.92) (*C*_R_80%). Since extrapolation outside the measured values would have been necessary for several participants to determine 70% of $\dot{V}\mathrm{O}_{2}\mathrm{peak}$ in accordance with Støa et al. (2020), 80% was chosen instead being always within the measuring range (except for two participants, which were excluded, see above) and giving the same *C*_R_ according to Helgerud et al. (2009). Independent of the work rate used, *C*_R_ was specified as oxygen cost in mL · kg$^{-1}$ ·﻿ m$^{-1}$ and as energy cost in $\hbox {J} \cdot \hbox {kg}^{-1} \cdot \hbox {m}^{-1}$ using the respiratory exchange ratio (Péronnet and Massicotte 1991) to take into account potential differences in substrate use (Barnes and Kilding 2015). In addition to *C*_R_, minute ventilation was determined at the three different running speeds.

Based on Eq. 1 (Støa et al. 2020; Støren et al. 2014), vLT2, as an indicator of endurance performance, was calculated using individual $\dot{V}\mathrm{O}_{2}\mathrm{peak}$, LT2_%_, and each of three differently determined values of *C*_R_ (using *C*_R_fix: _cal_LT2_fix_, using *C*_R_LT1: _cal_LT2_LT1_, using *C*_R_80%: _cal_LT2_80%_).

### Statistical analysis

The Statistical Package for the Social Sciences (version 27.0, IBM SPSS, Chicago, IL) was used for statistical analysis. All results were interpreted as significant for $\alpha =0.05$. For all data, normal distribution and homogeneity of variance were verified using the Shapiro-Wilk test and Levene’s test, respectively. Differences between male and female athletes were determined using independent sample *t*-tests.

Correlations between physiological parameters (model predictors and criteria) were determined using Pearson’s correlation coefficient *r*. These were interpreted as follows: < 0.30 $=$ negligible, 0.30–0.50 $=$ low, 0.50–0.70 $=$ moderate, 0.70–0.90 $=$ high, and > 0.90 $=$ very high (Mukaka 2012). Intra-class correlation coefficients (ICC) were calculated based on a single measure absolute agreement, two-way mixed model, to examine the agreement between the methods for determining *C*_R_ and between vLT2 and the modeled threshold estimates. According to Koo and Li (2016), the degree of agreement was interpreted as follows: < 0.50 $=$ poor, 0.50–0.75 $=$ moderate, 0.75–0.90 $=$ good, and > 0.90 $=$ excellent. In addition, a Bland-Altman analysis was performed to assess the concordance between vLT2 and the modeled threshold estimates.

Multiple regression analysis using bi-directional stepwise selection procedure (criteria: probability of *F* to enter $\le$ 0.05, probability of *F* to remove $\ge$ 0.10) was performed to estimate the association between vLT2 (dependent variable) and the three physiological variables $\dot{V}\mathrm{O}_{2}\mathrm{peak}$, LT2_%_, and *C*_R_ (independent variables). In addition, sex and age were included as independent variables to assess the influence of these anthropometric variables on the accuracy of the model.

To better understand the regression model, we additionally assessed the contributions of each predictor (independent variable) to the regression $R^2$ using a commonality analysis using R [R Core Team (2021), Version 4.1.2, *yhat* package] (Nathans et al. 2012; Ray-Mukherjee et al. 2014). In this way, it can be determined how much variance in the dependent variable is uniquely explained by a single predictor, independent of all other predictors (unique effects) and how much variance in the dependent variable is shared by a combination of the predictors (common effects). Further, negative commonality coefficients may indicate improved overall predictive power of the model associated with the suppressor variable, which removes some irrelevant variance or error in other variable(s) in the common effect, thus increasing the variance contributions of other variable(s) to the regression $R^2$ (Nathans et al. 2012; Pandey and Elliott 2010). All analyses were performed both for the whole sample and for the male and female subsets.

## Results

Table 1 summarizes the characteristics of the athletes. Both in absolute and relative terms, male athletes had a higher $\dot{V}\mathrm{O}_{2}\mathrm{peak}$ compared to female athletes ($p <0.001$). Regardless of the method used (in terms of both running speed and expression), male athletes showed poorer *C*_R_ ($p <0.001$) but better vLT2 ($p <0.001$) than female athletes. No sex difference was found for LT2_%_ ($p =0.999$). The running speeds corresponding to LT1 were 3.00 ± 0.33 $\hbox {m} \cdot \hbox {s}^{-1}$ and 2.70 ± 0.18 $\hbox {m} \cdot \hbox {s}^{-1}$, and those corresponding to 80% $\dot{V}\mathrm{O}_{2}\mathrm{peak}$were 3.29 ± 0.39 $\hbox {m} \cdot \hbox {s}^{-1}$ and 2.92 ± 0.22 $\hbox {m} \cdot \hbox {s}^{-1}$ in male and female athletes, respectively. In addition, minute ventilation was significantly higher in male than in female athletes at all submaximal running speeds used for *C*_R_ assessment (at LT1: 77.2 ± 14.6 $\hbox {L} \cdot \hbox {min}^{-1}$ vs. 63.2 ± 8.3 $\hbox {L} \cdot \hbox {min}^{-1}$, at 80% of $\dot{V}\mathrm{O}_{2}\mathrm{peak}$: 89.3 ± 19.8 $\hbox {L} \cdot \hbox {min}^{-1}$ vs. 70.9 ± 8.8 $\hbox {L} \cdot \hbox {min}^{-1}$, $p <0.001$), except at a running speed of 2.8 $\hbox {m} \cdot \hbox {s}^{-1}$ (108.6 ± 35.3 $\hbox {L} \cdot \hbox {min}^{-1}$ vs. 97.4 ± 26.0 $\hbox {L} \cdot \hbox {min}^{-1}$, $p = 0.069$).

**Fig. 2:**
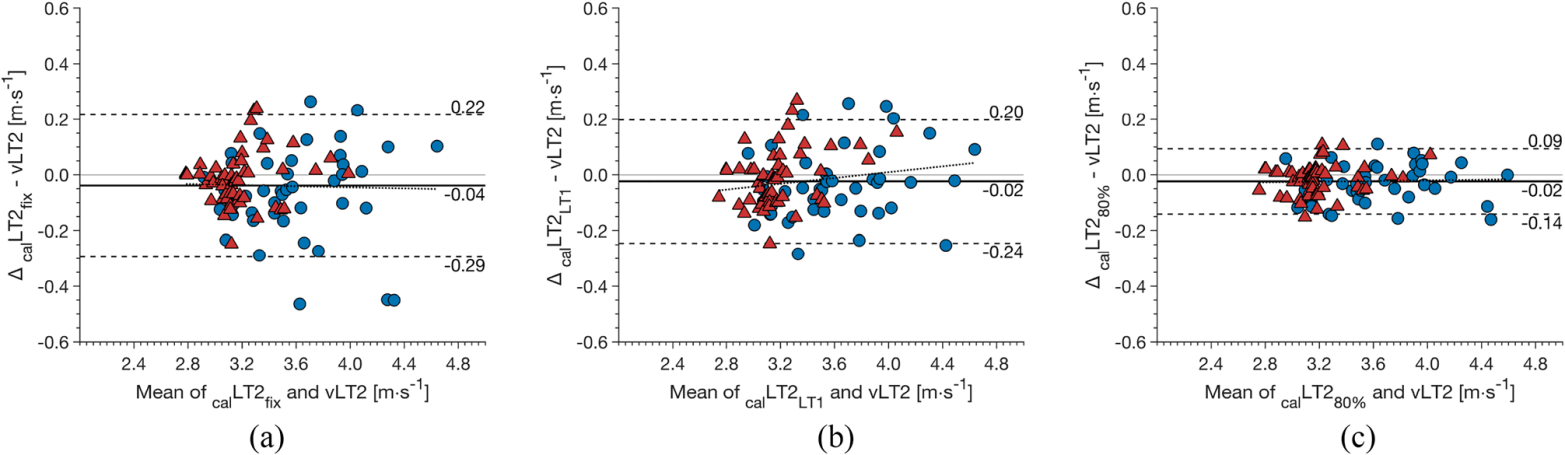
Bland-Altman Plots: differences between calculated running speed **a** at lactate threshold 2 determined by oxygen cost of running at a fixed running speed of 2.8 $\hbox {m} \cdot \hbox {s}^{-1}$ (_cal_LT2_fix_), **b** at lactate threshold 1 (_cal_LT2_LT1_), and **c** at 80% of maximal oxygen uptake (_cal_LT2_80%_) vs. running speed at lactate threshold 2 (vLT2) determined by modified maximal deviation method. The individual data of male (*N* = 45) and female (*N* = 55) athletes are presented by blue cycles and red triangles, respectively; the solid line indicates mean difference; the dashed lines indicate the limits of agreement (mean ± 1.96 standard deviation); the dotted line represents the fitted linear regression

The mean differences along with limits of agreement between _cal_LT2_fix_, _cal_LT2_LT1_, and _cal_LT2_80%_ vs. vLT2 are shown in the Bland-Altman plots (Fig. 2) and in Table 2. All modeled thresholds showed excellent concordance with vLT2 (Table 2).

**Table 2:** Mean difference (± limits of agreement) and intra-class correlation coefficients (ICC) between running speed at lactate threshold 2 (vLT2) vs. calculated lactate threshold 2 using the oxygen cost of running at a fixed running speed of 2.8 $\hbox {m} \cdot \hbox {s}^{-1}$ (_cal_LT2_fix_), at lactate threshold 1 (_cal_LT2_LT1_), and at 80% of maximal oxygen uptake (_cal_LT2_80%_)

	All (*N* = 100)		Males (*N* = 45)		Females (*N* = 55)	
	Mean difference [$\hbox {m} \cdot \hbox {s}^{-1}$]	ICC (95% CI)	Mean difference [$\hbox {m} \cdot \hbox {s}^{-1}$]	ICC (95% CI)	Mean difference [$\hbox {m} \cdot \hbox {s}^{-1}$]	ICC (95% CI)
_cal_LT2_fix_	– 0.04 ± 0.26	0.968 (0.950–0.979)	– 0.07 ± 0.32	0.955 (0.908–0.977)	– 0.02 ± 0.18	0.962 (0.934–0.978)
_cal_LT2_LT1_	– 0.02 ± 0.22	0.979 (0.968–0.986)	– 0.03 ± 0.25	0.976 (0.956–0.987)	– 0.01 ± 0.20	0.959 (0.929–0.976)
_cal_LT2_80%_	– 0.02 ± 0.12	0.993 (0.988–0.996)	– 0.02 ± 0.13	0.992 (0.983–0.996)	– 0.02 ± 0.11	0.986 (0.975–0.992)

*CI* confidence interval

Since *C*_R_80% provided the model with the highest accuracy due to the smallest limits of agreement (i.e., _cal_LT2_80%_ ± 4% vs. and _cal_LT2_fix_ ± 7% and ± 8%, respectively), it was used for further regression analyses. The relationships between vLT2 and the three physiological variables were presented both for the whole sample and separated by sex in Table 3.

**Table 3:** Model summary resulting from stepwise multiple regression analyses using the modeled running speed corresponding to lactate threshold 2 as dependent variable and classical physiological parameters as independent variables

Model	Variable	Beta	SE	Std. Beta	*t*	*p*	*r*	*R*^2^	Adj. *R*^2^	*F*	*p*
Analysis 1: All (*N* = 100)											
1	(Constant)	1.033	0.176		5.877	< 0.001		0.650	0.646	181.73	< 0.001
	$\dot{V}\mathrm{O}_{2}\mathrm{peak}$	0.046	0.003	0.806	13.481	< 0.001					
2	(Constant)	2.894	0.153		18.890	< 0.001		0.899	0.897	431.55	< 0.001
	$\dot{V}\mathrm{O}_{2}\mathrm{peak}$	0.063	0.002	1.113	29.378	< 0.001					
	Oxygen *C*_R_80%	– 12.409	0.802	– 0.586	– 15.471	< 0.001					
3	(Constant)	– 0.388	0.226		– 1.718	0.089		0.971	0.970	1086.10	< 0.001
	$\dot{V}\mathrm{O}_{2}\mathrm{peak}$	0.065	0.001	1.142	56.078	< 0.001	0.806				
	Oxygen *C*_R_80%	– 14.491	0.449	– 0.685	– 32.244	< 0.001	– 0.003				
	LT2_%_	0.042	0.003	0.283	15.585	< 0.001	0.175				
Analysis 2: Males (*N* = 45)											
1	(Constant)	0.625	0.389		1.606	0.116		0.581	0.571	59.63	< 0.001
	$\dot{V}\mathrm{O}_{2}\mathrm{peak}$	0.053	0.007	0.762	7.722	< 0.001					
2	(Constant)	3.076	0.325		9.473	< 0.001		0.876	0.871	149.03	< 0.001
	$\dot{V}\mathrm{O}_{2}\mathrm{peak}$	0.062	0.004	0.888	15.949	< 0.001					
	Oxygen *C*_R_80%	– 12.827	1.280	– 0.558	– 10.024	< 0.001					
3	(Constant)	– 0.407	0.369		– 1.105	0.276		0.967	0.965	402.69	< 0.001
	$\dot{V}\mathrm{O}_{2}\mathrm{peak}$	0.062	0.002	0.895	30.818	< 0.001	0.762				
	Oxygen *C*_R_80%	– 14.858	0.694	– 0.646	– 21.396	< 0.001	– 0.359				
	LT2_%_	0.045	0.004	0.313	10.643	< 0.001	0.170				
Analysis 3: Females (*N* = 55)											
1	(Constant)	1.588	0.277		5.737	< 0.001		0.390	0.379	33.92	< 0.001
	$\dot{V}\mathrm{O}_{2}\mathrm{peak}$	0.034	0.006	0.625	5.824	< 0.001					
2	(Constant)	2.806	0.191		14.665	< 0.001		0.813	0.806	112.92	< 0.001
	$\dot{V}\mathrm{O}_{2}\mathrm{peak}$	0.064	0.004	1.173	14.946	< 0.001					
	Oxygen *C*_R_80%	– 12.143	1.121	– 0.851	– 10.836	< 0.001					
3	(Constant)	– 0.177	0.274		– 0.649	0.519		0.949	0.946	318.67	< 0.001
	$\dot{V}\mathrm{O}_{2}\mathrm{peak}$	0.065	0.002	1.189	28.831	< 0.001	0.625				
	Oxygen *C*_R_80%	– 14.330	0.617	– 1.004	– 23.208	< 0.001	– 0.094				
	LT2_%_	0.039	0.003	0.396	11.725	< 0.001	0.283				

*Beta* beta coefficient, *SE* standard error, *Std. Beta* standardized beta coefficient, *r* correlation coefficient to the running speed at lactate threshold 2, $R^2$ coefficient of determination, *Adj*. $R^2$ adjusted coefficient of determination, $\dot{V}\mathrm{O}_{2}\mathrm{peak}$ maximal oxygen uptake, $C_{R}80\%$ cost of running determined at 80% of $\dot{V}\mathrm{O}_{2}\mathrm{peak}$, $LT2_{\%}$ fractional utilization of $\dot{V}\mathrm{O}_{2}\mathrm{peak}$

Entering $\dot{V}\mathrm{O}_{2}\mathrm{peak}$, LT2_%_, and *C*_R_80% into the multiple linear regression model was able to explain 97%, 97%, and 95% of the variance in vLT2 for all, male, and female athletes, respectively (Table 3). Based on the selection criteria in the stepwise selection procedure, sex and age were not included in the final multiple regression model.

**Fig. 3:**
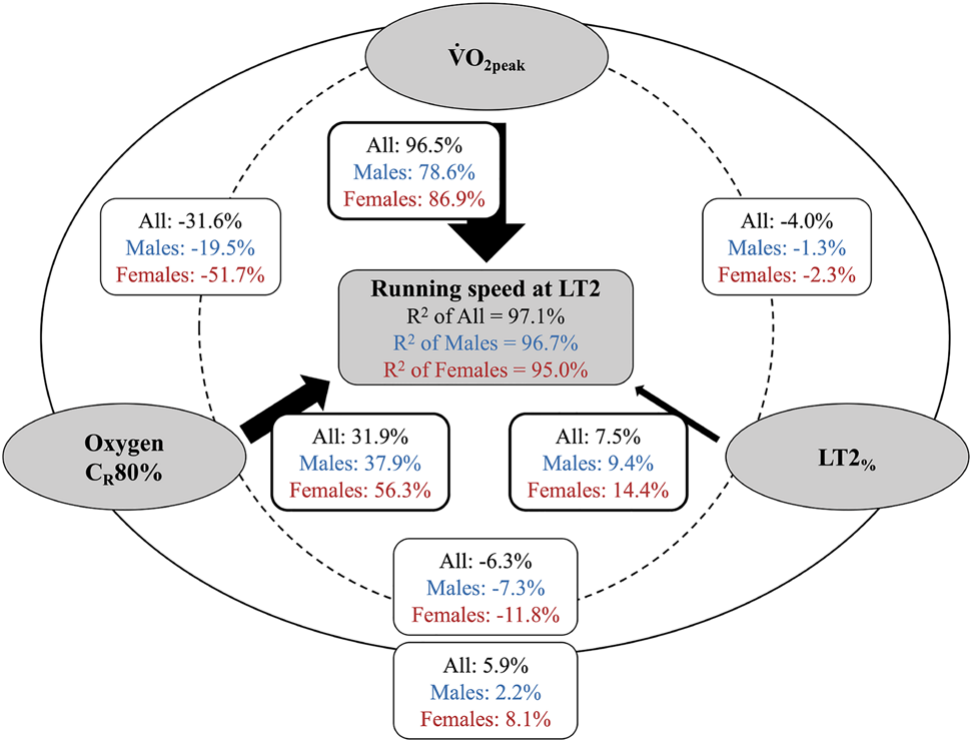
Graphical summary of commonality analyses for the modeled running speed at lactate threshold 2 (LT2) within all (*N* = 100), male (*N* = 45) and female (*N* = 55) athletes. The percentage contribution of each unique predictor to the total regression effect (i.e., $R^2$) is presented by the black filled arrows; the dashed lines and external solid lines represent the common effects of two and all predictors in $R^2$, respectively. $\dot{V}\mathrm{O}_{2}\mathrm{peak}$: maximal oxygen uptake; *C*_R_80%: cost of running determined at 80% of $\dot{V}\mathrm{O}_{2}\mathrm{peak}$; LT2_%_: fractional utilization of $\dot{V}\mathrm{O}_{2}\mathrm{peak}$ at LT2.

According to the commonality analysis, $\dot{V}\mathrm{O}_{2}\mathrm{peak}$ showed the highest unique contribution to the total regression $R^2$ followed by *C*_R_80% and LT2_%_ regardless of the analyzed subset. Regarding the common effects, all sets of predictors showed a negative commonality coefficient, indicating the presence of suppression effects. The most noticeable suppression effect was observed between $\dot{V}\mathrm{O}_{2}\mathrm{peak}$ and *C*_R_80%, which was at – 31.6% in the whole sample. This was more pronounced in the female (– 51.7%) compared to the male subset (– 19.5%). The detailed results of the commonality analyses are depicted in Fig. 3.

## Discussion

The present study investigated the accuracy of the classical model with the physiological variables (i.e., $\dot{V}\mathrm{O}_{2}\mathrm{peak}$, LT2_%_, and *C*_R_), applied in previous studies (McLaughlin et al. 2010; Støa et al. 2020; Støren et al. 2014), in determining vLT2 as an indicator of endurance performance in young squad athletes of different disciplines, ages, and sexes. We found an excellent accordance between the modeled and experimentally determined vLT2 (ICC $\ge$ 0.959) with a very low systematic bias (mean difference ± limits of agreement $\le$ 0.07 ± 0.32 $\hbox {m} \cdot \hbox {s}^{-1}$) independent of sex and age, supporting the applicability of _cal_LT2 to assess aerobic endurance performance also in young athletes. Furthermore, the accuracy of vLT2 modeling was improved, when *C*_R_ was determined by individualized approaches (especially *C*_R_80%) rather than at a fixed speed (i.e., *C*_R_fix) (Fig. 2). According to the stepwise regression and commonality analyses, $\dot{V}\mathrm{O}_{2}\mathrm{peak}$ is the most important factor for vLT2 in both sexes, followed by *C*_R_, whereas LT2_%_ has the least influence (Fig. 3).

In the present investigation, *C*_R_ determined by different methods showed very high similarity with each other (Table 1). However, the application of individualized approaches, particularly *C*_R_80%, further improved the accuracy of the model for calculating vLT2 as reflected by the lower variation (i.e., limits of agreement) compared to the other methods assessing *C*_R_ (Table 2 and Fig. 2). Especially in a heterogeneous sample as in our study, measuring $\dot{V}\mathrm{O}_{2}$ at a fixed running speed (i.e., *C*_R_fix) results in different metabolic responses (e.g., percent utilization of $\dot{V}\mathrm{O}_{2}\mathrm{peak}$ and substrate utilization) and thus impairs the inter-individual comparability of *C*_R_. This might have contributed to the lower accuracy of _cal_LT2_fix_ compared to the other methods (i.e., larger random variation). To compensate for inter-individual variability in metabolic response, we suggested to assess *C*_R_ at a specific submaximal metabolic anchor, i.e., LT1. However, incorporating *C*_R_LT1 in the model (i.e., _cal_LT2_LT1_) did not considerably improve its accuracy in calculating vLT2 as compared to *C*_R_80%. This result can be explained in part by the fact that the running speed at LT1 (2.84 ± 0.30 $\hbox {m} \cdot \hbox {s}^{-1}$) was similar to 2.8 $\hbox {m} \cdot \hbox {s}^{-1}$ (running speed for *C*_R_fix) and significantly lower than that at 80% of $\dot{V}\mathrm{O}_{2}\mathrm{peak}$ (3.09 ± 0.36 $\hbox {m} \cdot \hbox {s}^{-1}$). Although it is now widely established that *C*_R_ is independent of the respective running speed (Di Prampero et al. 2009; Shaw et al. 2014), there are still conflicting findings indicating an increased or decreased *C*_R_ with the running speed (Iaia et al. 2009; Jones and Doust 1996; Lacour and Bourdin 2015; Svedenhag and Sjödin 1994). Assessing *C*_R_ at high relative running speed is likely to represent typical movement patterns (e.g., stride length and frequency) and to consider the individual metabolic demand, especially near to LT2. In this regard, it can be speculated that the running speed at 80% of $\dot{V}\mathrm{O}_{2}\mathrm{peak}$ may have better represented the inter-individual variation in running energetics and/or mechanics near LT2 compared to the running speed at LT1, thereby increasing the accuracy of the computational model. This is supported by the previous suggestion of Lacour and Bourdin (2015) that athletes’ performance may be more accurately predicted from *C*_R_ determined at high relative running speed.

Regarding the criterion, Støren et al. (2014) and Støa et al. (2020) used a threshold concept with a warm-up level of blood lactate concentration plus a fixed absolute value (2. 3 $\hbox {mmol} \cdot \hbox {L}^{-1}$) to determine LT2 as an estimate of maximal metabolic steady state. Although such an approach has been repeatedly used in previous studies as an established indicator of endurance performance [e.g., Helgerud et al. (1990, 2009)], there is no explicit study assessing its systematic bias and absolute agreement compared to the underlying physiological concept of a maximal metabolic steady state apart from unpublished work [i.e., Helgerud et al. (1990)], which is crucial for ensuring the validity of a threshold concept (Faude et al. 2009). In addition, the increase in blood lactate concentrations of a certain fixed value might not always be equally meaningful, since it is highly affected by various factors (e.g., test protocols, training- and nutrition-status) (Svedahl and MacIntosh 2003). In contrast, we applied a mathematical model for determining inflection points as a determination criteria for LT2, which is based on blood lactate kinetics rather than absolute concentrations (Zwingmann et al. 2019). Even though the use of mathematical models for LT2 determination has been criticized by some authors because of the lacking physiological fundamental (Janeba et al. 2010), its validity for estimating the maximal lactate steady state has been verified by systematic analyses (Jamnick et al. 2018; Zwingmann et al. 2019).

In the present study, the stepwise regression analysis showed that 97% of the total variance (males: 97%, females: 95%) in vLT2 in young squad athletes of different disciplines was explained by $\dot{V}\mathrm{O}_{2}\mathrm{peak}$, LT2_%_, and *C*_R_, supporting that these are the three primary physiological factors influencing aerobic endurance performance (McLaughlin et al. 2010). The single most important determinant of vLT2 independent of sex was $\dot{V}\mathrm{O}_{2}\mathrm{peak}$, which is in accordance with previous research (McLaughlin et al. 2010; Støa et al. 2020; Støren et al. 2014), emphasizing the importance of aerobic energy supply during prolonged weight-bearing exercise such as running. Therefore, especially in heterogeneous samples as in the present study, endurance performance is strongly related to $\dot{V}\mathrm{O}_{2}\mathrm{peak}$. Likewise, the lower values observed in the young female compared to the male athletes are in accordance with previous studies and are likely related to body composition (i.e., greater percentage of body fat) and oxygen carrying capacity (i.e., lower hematocrit levels) (Besson et al. 2022).

Based on the commonality analysis, *C*_R_, being the second major factor influencing vLT2, seems to act as a suppressor which purifies the “irrelevant” variance of other independent variables (i.e., negative common effects), and thus improves their contribution to the regression model. In particular, the pronounced suppression effect of *C*_R_ in combination with $\dot{V}\mathrm{O}_{2}\mathrm{peak}$ (– 19.5% to – 51.7% of total $R^2$) can emphasize the crucial role of the interaction between maximal aerobic capacity and movement economy for endurance performance (Joyner and Coyle 2008). Indeed, in the present investigation, *C*_R_ separately exhibited only negligible to low correlation with vLT2 (*r* = – 0.359 to – 0.003), but its incorporation into the regression model in addition to $\dot{V}\mathrm{O}_{2}\mathrm{peak}$ resulted in a significantly improved $R^2$ with a significant (standardized) beta weight in both the whole group and the male and female subgroups (see Table 3). Furthermore, the product of $\dot{V}\mathrm{O}_{2}\mathrm{peak}$ and *C*_R_ (expressing maximal aerobic speed) has been demonstrated to be a very good predictor for 16-km time trial performance ($R^2$ = 0.94) and vLT2 ($R^2$ = 0.85) in competitive runners (McLaughlin et al. 2010; Støa et al. 2020). Interestingly, the unique (56.3% vs. 37.9%) and common effects (– 51.7% vs. – 19.5%) of *C*_R_ on the total $R^2$ were distinctly higher in female than in male athletes implying the greater impact of *C*_R_ in determining endurance performance in females. Further, female athletes showed lower *C*_R_ values independent of the determination method. This is an interesting finding which contributes to the debate whether or not there are sex differences regarding *C*_R_(Besson et al. 2022). While some authors argue that sex differences in *C*_R_ disappear when expressed as relative intensities (i.e., as percentage of $\dot{V}\mathrm{O}_{2}\mathrm{peak}$ or lactate threshold) (Fletcher et al. 2013; Helgerud et al. 1990), our data indicate that, at least in young athletes, females exhibit lower values whether expressed as absolute (*C*_R_fix) or relative (*C*_R_80% and *C*_R_LT1) values.  Besides differences in anthropometric dimensions (e.g., body height, see Table 1), the lower *C*_R_ in female athletes might be due to neuromuscular characteristics of the lower extremities such as lower-body stiffness or Achilles moment arm length as shown for well-trained distance runners (Barnes et al. 2014). Furthermore, since we observed significantly higher minute ventilation at the speed corresponding to 80% of $\dot{V}\mathrm{O}_{2}\mathrm{peak}$ in male compared to female athletes, it can be assumed that the differences in *C*_R_ may be attributed, at least in part, to increased demands of breathing. Thus, it has been shown earlier that running economy was improved by training induced decrease in minute ventilation (Franch et al. 1998). Nonetheless, it should be noted that our study did not take into account hormonal changes related to the phase of the menstrual cycle in female athletes, which is known to affect *C*_R_ (Besson et al. 2022; Dokumacı and Hazır 2019).

Besides sex-specific differences in *C*_R_, it is unclear why *C*_R_ had a greater impact on vLT2 determination in young female athletes than in males. However, beside the aforementioned factors (e.g., anthropometric, neuromuscular, and cardio-respiratory), there are sex-specific differences related to substrate oxidation during exercise and muscle tissue characteristics (e.g., proportion of type I muscle fibers and muscle capillarization), that could affect male and female athletes differently in terms of submaximal energy metabolism (Besson et al. 2022). These differences may partly explain why *C*_R_ appears to have a stronger influence on endurance performance in young female compared to male athletes, similar to the previous findings of Støa et al. (2020) in adult runners. In this context, future longitudinal studies might investigate whether female athletes can actually profit more than male athletes from an improvement in *C*_R_.

In contrast to $\dot{V}\mathrm{O}_{2}\mathrm{peak}$ and *C*_R_, we found no sex difference in LT2_%_ (87.0 ± 2.9% vs. 87.0 ± 2.4%) with a very low inter-individual variation (coefficient of variation $=$ 3%). These results are in line with previous investigations indicating no difference in LT2_%_ between well-trained male and female runners (McLaughlin et al. 2010) as well as between elite, national, and recreational runners (Støa et al. 2020). Further, the LT2_%_-values in the previous studies with adult recreational and elite runners (72–93%) are in a similar range to those in the current study (80–94%). Taken together, it seems plausible to assume that LT2_%_ does not vary substantially depending on the aerobic endurance level. Moreover, the minor contribution of LT2_%_ to the regression model for determining vLT2 in the present study (Fig. 3) provides further support for the assumption that LT2_%_ is not a major factor affecting aerobic endurance performance (McLaughlin et al. 2010; Støa et al. 2020; Støren et al. 2014). Nonetheless, further studies need to investigate whether long-term adaptations of LT2_%_ lead to altered aerobic endurance performance (i.e., vLT2) in young athletes of different disciplines.

Due to the high accuracy in estimating vLT2, the proposed model allows to draw conclusions about the limiting factors (mainly $\dot{V}\mathrm{O}_{2}\mathrm{peak}$ and *C*_R_) of endurance performance of young athletes of various disciplines and both sexes, and may therefore guide future training design. Since total time to improve endurance capacity in technically or tactically demanding sports is limited especially in young athletes with a restricted schedule, this time needs to be utilized as efficiently as possible. Thus, depending on the individual physiological prerequisites, training prescription may focus either on improving $\dot{V}\mathrm{O}_{2}\mathrm{peak}$ for example using high intensity interval training or on improving *C*_R_ for example by implementing explosive- and maximal-strength training (Rønnestad and Mujika 2013; Støa et al. 2020). Future studies should use the predictors to model endurance performance longitudinally, e.g., over one or multiple seasonal cycles to examine whether training induced changes in the physiological predictors actually lead to the intended changes in endurance performance.

## Conclusion

In conclusion, the classical model using $\dot{V}\mathrm{O}_{2}\mathrm{peak}$, *C*_R_, and LT2_%_ to determine vLT2 is also suitable for assessing endurance performance in young squad athletes of different disciplines, ages, and sexes. The accuracy of the model was further improved by an individual determination of *C*_R_ (in particular *C*_R_80%). $\dot{V}\mathrm{O}_{2}\mathrm{peak}$ and *C*_R_ were found to have the most important contributions in determining vLT2. While in young female athletes, the impact of *C*_R_ on endurance performance appeared to be greater than that in male athletes, LT2_%_ was generally found to have the least impact on vLT2 determination.

## Abbreviations

_cal_LT2: Calculated speed corresponding to lactate threshold 2
_cal_LT2_fix_: Calculated speed corresponding to lactate threshold 2 determined using cost of running determined at a fixed speed of 2.8 $\hbox {m} \cdot \hbox {s}^{-1}$
_cal_LT2_LT1_: Calculated speed corresponding to lactate threshold 2 determined using cost of running determined at lactate threshold 1
_cal_LT2_80%_: Calculated speed corresponding to lactate threshold 2 determined using cost of running determined at 80% of maximal oxygen uptake
*C*: Cost of movement
*C*_R_: Cost of running
*C*_R_fix: Cost of running determined at a fixed speed of 2.8 $\hbox {m} \cdot \hbox {s}^{-1}$
*C*_R_LT1: Cost of running determined at lactate threshold 1
*C*_R_80%: Cost of running determined at 80% of maximal oxygen uptake
LT1: Lactate threshold 1 (first rise in blood lactate concentration)
LT2_%_: Fractional utilization of maximal oxygen uptake at lactate threshold 2
ICC: Intra-class correlation coefficient
LT2: Lactate threshold 2
$\dot{V}\mathrm{O}_{2}\mathrm{peak}$: Maximal oxygen uptake
$$\dot{V}\mathrm{O}_{2}$$: Oxygen uptake
vLT2: Running speed corresponding to lactate threshold 2
